# Defining Dystonic Tremor

**DOI:** 10.2174/157015913804999478

**Published:** 2013-01

**Authors:** Rodger J Elble

**Affiliations:** Department of Neurology, Southern Illinois University School of Medicine, 751 North Rutledge, PO Box 19643, Springfield, IL 62794-9643, USA

**Keywords:** Dystonia, essential tremor, tremor.

## Abstract

A strong association between dystonia and tremor has been known for more than a century. Two forms of tremor in dystonia are currently recognized: 1) dystonic tremor, which is tremor produced by dystonic muscle contraction and 2) tremor associated with dystonia, which is tremor in a body part that is not dystonic, but there is dystonia elsewhere. Both forms of tremor in dystonia frequently resemble essential tremor or another pure tremor syndrome (e.g., isolated head and voice tremors and task-specific writing tremor), and relationships among these tremor disorders have long been debated. Misdiagnosis is common because mild dystonia is frequently overlooked in patients with tremor. It is now clear that essential tremor is a syndrome, not a specific disease, and the use of essential tremor as a specific clinical diagnosis is arguably an impediment to elucidating this and other pure tremor syndromes and their relationship to dystonia. A new classification, primary tremor, is proposed and would be used for any disorder in which tremor is the sole or principal abnormality with no identifiable etiology other than possible genetic inheritance. This classification scheme would facilitate tremor research by moving the focus from the narrow question “Is it essential tremor?” to a broader consideration of what genetic and environmental factors cause primary tremor disorders, and how do they relate to dystonia and other neurological disorders.

## TREMOR AND DYSTONIA 

An association between tremor and dystonia has been recognized for more than 100 years [1]. Yanagisawa and Goto [2] performed a multichannel electromyographic (EMG) analysis of dystonic muscle contractions and found that phasic muscle contraction was common. Rhythmic 5-11 Hz bursts of EMG were seen in voluntary contractions, and rhythmic and irregular bursts of EMG at 1-6.5 Hz occurred in the involuntary dystonic contractions. Whether some of these phasic muscle contractions are sufficiently rhythmic to be called tremor can be debated, and the tremor in dystonic contractions is widely acknowledged to be irregular in rhythm and amplitude [3]. However, dystonic tremor and the tremor in voluntary contractions of neighboring muscles with no apparent dystonia can be so rhythmic that it is mistaken for classic essential tremor (ET) [4-6].

In keeping with these observations, the Consensus Statement of the Movement Disorder Society (MDS) on tremor [7] recognized two types of tremor in dystonia patients: 1) dystonic tremor, which is tremor in a dystonic body part (i.e., dystonic muscle contraction), and 2) tremor associated with dystonia, which is tremor in a body part with no dystonia, but the patient has dystonia elsewhere. The MDS Consensus Statement also acknowledged that tremor can be the only neurologic sign in patients carrying a gene for dystonia (“dystonia gene-associated tremor”).

Tremor is so common in primary dystonia that it is considered part and parcel of primary dystonia [8]. Dystonic tremor can occur at rest, in sustained postures, and in voluntary movement, and it may also be focal and task specific [5, 9, 10]. These characteristics are similar to those of ET and other less common focal and task specific tremors (e.g., isolated head and voice tremor and task-specific writing tremor) [7]. 

Tremor associated with dystonia is particularly controversial from the standpoint of its relationship to ET [11-13]. This controversy is difficult to address because dystonia is a heterogeneous movement disorder, not a specific disease, and the same is arguably true for ET [14].

## ESSENTIAL TREMOR 

In the late 1800s, physicians recognized a common condition in which abnormal tremor occurred primarily in the upper limbs, in the absence of other neurologic signs, and they called this condition essential tremor to emphasize that tremor was the very essence of this disorder [15]. This view of ET as a pure or monosymptomatic tremor disorder persisted throughout the 1900s [16] and is embodied in the currently used diagnostic criteria for ET (Table **1**) [7, 17].

Differences exist among the published clinical criteria for ET (Table **1**). The MDS consensus criteria include isolated head tremor in the absence of dystonic posturing as a variant of ET [7]. By contrast, the Tremor Investigation Group (TRIG) criteria exclude isolated head tremor and require at least a 5 year history of hand tremor [18]. These differences notwithstanding, classic ET remains a narrowly defined disorder consisting of action tremor in the hands and commonly in the head and voice, with little or no tremor in the lower limbs and torso [19, 20]. Other neurologic signs must be absent. The diagnosis of ET is based solely on clinical exam and neurological history. There is no biological marker or diagnostic test.

Researchers have debated for decades whether ET is a specific disease or a common clinical presentation (i.e., syndrome or phenotype) of multiple different diseases. For the following reasons, it seems certain that ET is not a specific disease:

1. Data from epidemiologic studies suggest that ET is a phenotype of multiple disorders [21]. People with the clinical characteristics of ET are common in families with hereditary dystonia [12, 22]. An epidemiologic study in Spain revealed an increased risk of dementia in older ET patients (age > 64) if their tremor developed after age 65 but not in those who developed tremor earlier in life [23, 24]. There was also an increased incidence of Parkinson disease (PD) in this cohort of older ET patients [25].
2. Some postmortem studies have identified two distinct neuropathologic abnormalities: brainstem Lewy body pathology and cerebellar Purkinje cell loss [26]. There are probably additional patients with no, or as yet unknown, microscopic pathology [27-29].
3. There appear to be multiple genes that cause hereditary ET, and there are probably also multiple genetic risk factors that play a role in “sporadic” ET [30-32]. It is unclear why only one relatively minor risk factor gene (LINGO1) [31] has been found despite many credible linkage studies of large families and genome wide association studies of large patient cohorts. Rich genetic heterogeneity, polygenic inheritance, and high prevalence of ET risk genes are possible reasons for our inability to find more genes. Large families could contain multiple tremor genes that are bred into these families over multiple generations, making the pattern of genetic inheritance difficult to discern and the identity of these genes difficult to define. Another reason may be that the clinical criteria for ET are too narrowly defined. A similar problem existed in the initial genetic studies of PD, where a too narrowly defined phenotype for PD resulted in a failure to find concordance between identical twins [33]. We now know that a single Parkinson gene mutation can produce substantially different signs and symptoms in patients with the same mutation, even in the same family [34]. The same is true for the gene mutations causing hereditary ataxias [35] and hereditary dystonias [36], and the same is probably true for hereditary ET.

While some of these observations are still controversial (e.g., the postmortem findings in ET [28, 37]), it is undeniable that classic ET is a clinical tremor syndrome with multiple etiologies, including dystonia. Nevertheless, ET is often viewed, studied and managed as though it were a specific disease. This artificially narrow view of ET arguably impedes the quest for better treatment and genetic elucidation [38, 39].

The definition of ET as a pure tremor syndrome places great emphasis on the exclusion of other neurologic signs. Clinicians commonly miss or dismiss as insignificant other neurologic signs in tremor patients, resulting in the misdiagnosis of ET [4, 6]. Patients diagnosed with ET commonly exhibit subtle abnormal body postures that could be dystonia [5]. Such postures are diagnosed as dystonia in dystonia studies [13], but similar postures are often dismissed as insignificant or compensatory (to reduce tremor) in ET studies [5, 40]. In other words, clinicians appear to be biased by the context in which these conditions are diagnosed. Dystonia is widely under-recognized [40] and is much more common than originally believed [41], and there is good reason to believe that ET is overdiagnosed [6]. Dystonic contractions can be so mild, focal or task-specific that they are overlooked by physicians, resulting in the misdiagnosis of ET, and tremor associated with focal and segmental dystonia can look so much like ET that researchers frequently ask whether this tremor is ET or a dystonic tremor [12, 13].

Meanwhile, the traditional definition of ET as a monosymptomatic disorder is being challenged due to increasing evidence that ET is associated with other signs and symptoms [38]. Thus, there is a trend among researchers to move away from the concept that ET is a monosymptomatic disorder. Movement disorder specialists have not been consistent in the criteria used in diagnosis of ET [42]. ET is losing its original meaning and is arguably no longer a valid clinical diagnosis. 

Further uncertainty stems from the relationship of ET with other pure tremor syndromes: task- or position-specific tremors such as primary writing tremor, isolated head tremor, and isolated voice tremor [7]. Patients with these less common tremor syndromes frequently have subtle or questionable signs of dystonia or may eventually develop unequivocal dystonia [43-45], and patients with these conditions may coexist with classic ET in the same family [46].

## TREMOR-DYSTONIA DIAGNOSTIC UNCERTAINTIES 

It is clear that primary dystonias can present as pure action tremor [5, 47]. It is also clear that dystonic posturing can be so mild that it is overlooked or interpreted as compensatory posturing or as some other condition [5, 13, 40]. Focal and segmental dystonias are often associated with ET-like tremor, and there is currently no laboratory, radiologic or neurophysiologic method of confidently distinguishing patients with classic ET from patients with subtle dystonia and ET-like tremor in the upper limbs, head or voice.

Dystonic tremor is often suppressed by sensory tricks (geste antagoniste) and may exhibit null points (body positions with no tremor), irregular jerky rhythm and amplitude, task or position specificity, persistence at rest, and overflow to neighboring body segments [3]. However, objective, quantitative criteria for these characteristics do not exist. Using accelerometry, Masuhr and coworkers quantified the suppression of head tremor in response to sensory tricks and found no effect from similar maneuvers in patients with ET [48]. However, the specificity and sensitivity of this approach were not determined, and the authors acknowledged the lack of a definitive diagnostic test for distinguishing dystonic head tremor from ET. Transcranial magnetic cortical stimulation and spinal Hoffman-reflex (H-reflex) studies have revealed statistically significant reductions in cortical inhibition and reciprocal H-reflex inhibition in dystonic patients compared to controls, but comparable abnormalities also have been found in psychogenic dystonia [49, 50], and differences between dystonic and non-dystonic patients are not sufficient for the diagnosis of dystonia [22, 51].

## A NEW TREMOR CLASSIFICATION SCHEME 

A new classification scheme for tremor is needed, one that emphasizes the complete and unbiased characterization of tremor and associated signs and symptoms in each patient. In this new classification scheme, the term primary (not essential) tremor would be used for any patient in which tremor is the sole or principal abnormality and there is no identifiable etiology other than possible genetic inheritance. With this classification, the emphasis would shift from the diagnosis (definition) of ET to the careful characterization of idiopathic tremor and all associated signs and symptoms. ET would no longer be a specific diagnosis or tremor classification although, if desired, patients with any definition of ET (e.g., TRIG or MDS criteria) could be extracted from a cohort of patients with primary tremor. A primary tremor classification scheme would greatly reduce the contentious debate regarding the relationship of ET to other disorders because the debate is changed from “Is the tremor ET?” to “What are the causes of primary tremor?”

Primary tremors would be classified as rest, postural, kinetic, intention, task-specific, and position-specific. The subsequent observation of other signs such as focal dystonia would not change the classification but would simply aid in clarifying the underlying etiology. Unlike the diagnosis of ET, the diagnosis of primary tremor is an acknowledgment that a specific diagnosis or etiology is still lacking. There is no assumption of a specific phenotype, so there is no pressure to dismiss associated signs (e.g., subtle or questionable dystonia) and symptoms as insignificant. 

## CONCLUSIONS 

A broad net must be cast to capture the information needed to fully understand the many diseases and genetic traits that cause primary tremor of the ET type and other less common types of primary tremor (e.g., focal and task-specific tremors). The proposed primary tremor classification scheme is less presumptuous than existing classification schemes that emphasize ET as a specific and narrowly defined disorder. This new classification should enhance our search for tremor-producing genes and better treatment and should facilitate the elucidation of tremor-dystonia relationships.

## Figures and Tables

**Table 1.** Diagnostic Criteria for Classic (Definite) Essential Tremor

Tremor Investigation Group Criteria [18]	Movement Disorder Society Consensus Criteria [7]
Inclusion criteria: Bilateral postural tremor, with or without kinetic tremor, in the hands that is visible and persistent Duration longer than 5 years	Inclusion criteria: Bilateral, largely symmetric postural or kinetic tremor of the hands that is visible and persistent Additional or isolated head tremor in the absence of abnormal posturing
Exclusion criteria: Other abnormal neurologic signs (with the exception of the presence of tremor and Froment’s sign. The full neurologic examination should be normal for age) Presence of known causes of enhanced physiologic tremor Concurrent or recent exposure to tremorogenic drugs or the presence of a drug withdrawal state Direct or indirect trauma to the nervous system within 3 months preceding the onset of tremor Historic or clinical evidence of psychogenic origins of tremor Convincing evidence of sudden onset or evidence of stepwise deterioration	Exclusion criteria: Other abnormal neurologic signs, especially dystonia The presence of known causes of enhanced physiologic tremor, including current or recent exposure to tremorogenic drugs or the presence of a drug withdrawal state Historic or clinical evidence of psychogenic tremor Convincing evidence of sudden onset or evidence of stepwise deterioration Primary orthostatic tremor Isolated voice tremor Isolated position-specific or task-specific tremors, including occupational tremors and primary writing tremor Isolated tongue or chin tremor Isolated leg tremor
